# Quality of Dry-Cured Ham from Entire, Surgically and Immunocastrated Males: Case Study on Kraški Pršut

**DOI:** 10.3390/ani10020239

**Published:** 2020-02-03

**Authors:** Marjeta Čandek-Potokar, Martin Škrlep, Eliza Kostyra, Sylwia Żakowska-Biemans, Klavdija Poklukar, Nina Batorek-Lukač, Kevin Kress, Ulrike Weiler, Volker Stefanski

**Affiliations:** 1 Agricultural Institute of Slovenia, Hacquetova ul. 17, 1000 Ljubljana, Slovenia; martin.skrlep@kis.si (M.Š.); klavdija.poklukar@kis.si (K.P.); nina.batorek@kis.si (N.B.-L.); 2 Institute of Human Nutrition Sciences, Warsaw University of Life Sciences (WULS-SGGW), ul. Nowoursynowska 159c, 02-787 Warsaw, Poland; eliza_kostyra@sggw.pl (E.K.); sylwia_zakowska_biemans@sggw.pl (S.Ż.-B.); 3 Department of Behavioral Physiology of Livestock, Institute of Animal Science, University of Hohenheim, Garbenstraße 17, 70599 Stuttgart, Germany; kress.kevin@uni-hohenheim.de (K.K.); weiler@uni-hohenheim.de (U.W.)

**Keywords:** immunocastration, entire male, castration, pig, dry-cured ham

## Abstract

**Simple Summary:**

The initiative to stop the surgical castration of piglets calls for the assessment of alternative solutions. The issue is particularly sensitive for the processing of traditional products. This study demonstrated important differences between male sex categories and showed that dry-cured ham from entire males presented distinct sensory depreciation and also differed in many other aspects important in dry-cured ham production. Under the conditions of the present study, i.e., standard slaughter age and weight and delay between immunocastration and slaughter, dry-cured ham from surgical castrates showed the most distinct properties, while immunocastrated pigs were more similar in many aspects to entire males, however, their main advantage was in their sensory attributes, i.e., absence of boar taint.

**Abstract:**

Alternative solutions to the surgical castration of piglets need to be assessed because this is a particularly sensitive issue for the processing of traditional pork products. Currently, the available information about the advantages and drawbacks of castration for dry-cured products is limited; thus, the objective of this study was to evaluate the quality of Slovenian dry-cured ham (Kraški pršut) from entire males (EM), immunocastrates (IC) and surgical castrates (SC). Hams (12 per sex group) were processed for one year and physical-chemical, rheological and sensory analysis of the dry-cured hams was performed. With regard to processing aptitude, the main difference was in the subcutaneous fat thickness, which influenced the level of dehydration and salt intake. This was further reflected in the physical-chemical traits and the texture, which were measured instrumentally or assessed by panelists. Regarding the aforementioned traits, EM and IC were generally similar and different from SC. On the contrary, sensory profiling of odor, taste and flavor demonstrated that EM had the lowest overall sensory quality, different from both IC and SC, and presented odors and flavors described as sweat, manure, sharp and persistent. We confirmed that dry-curing did not eliminate the perception of boar taint in the product from EM. The IC were similar in many aspects to EM except for the odor, taste and flavor of dry-cured hams, in which case they were more similar to SC.

## 1. Introduction

In pig production, it is a widespread practice to castrate male piglets in order to prevent an unpleasant off-flavor, the so-called boar taint, which can appear in the meat of sexually mature entire males. EU legislation allows the castration of male piglets without anesthesia/analgesia in the first seven days of life; however, this practice has been criticized from the animal welfare point of view [1]. Thus, in recent years, the European pig industry sector has considered ending this practice [2,3]. However, before this can happen, certain problems and alternative solutions must be assessed, not only because of the risk of boar taint but also due to the potential deterioration in the technological quality of the meat and its consequences for processed products needs to be evaluated [4]. The challenges are particularly relevant to traditional products, where the highest risks are associated with fat quantity and quality [5]. Boar taint (an unpleasant aroma attributed to the presence of two malodorous compounds, androstenone and skatole) is more apparent when fat content is high, no masking ingredients are used and the product is consumed warm [6]. It has been detected in dry-cured products, even if these were not consumed warm [7,8]. According to Tørngren et al. [9], androstenone levels must be below 0.4 ppm and the serving temperature must be below 23 °C for consumers to not detect it in the processed pork. A review of consumer studies [6] indicated the need for a better understanding of the risks related to the perception of boar taint in the case of different product types. However, there are not many studies available that compare the alternatives to surgical castration with regard to the processing aptitude of the meat and dry-cured product quality. The quality of the raw material is very important in dry-cured ham processing and is affected by many factors, including the sex of pigs [10]. Thus, the aim of the present study was to compare the effects of three male sex categories (immunocastrates, surgical castrates and entire males) on the production of dry-cured ham, more precisely, Kraški pršut, a Slovenian dry-cured ham that has protected geographical indication (PGI) status. Its manufacture is clearly defined with specific demands regarding the raw material (green ham weight, visual appearance, pH, fat thickness), and the minimum processing weight loss. A minimum curing period of 12 months is prescribed [11]. For this product, as is the case for many other similar PGI or unbranded dry-cured ham products, the origin of the raw material is not prescribed and provisions made with raw hams stem from standard pig production. In such situations, the probability of getting green hams from different castration methods is higher and raw material variability is increased. Due to differences in the physiological and metabolic characteristics of the three sex types of pigs, the raw material properties, i.e., its seasoning aptitude, may be altered. The question of interest is what are the consequences for the final product quality and the adaptations in processing that may be required. The present study covers the assessment of the raw material properties, processing yield and final product quality (chemical, rheological and sensory quality traits) as affected by the male sex group of pigs.

## 2. Materials and Methods

### 2.1. Origin and Processing of Hams

The hams for this study originate from a wider study evaluating immunocastration (for more details see [12]). We compared hams from surgical castrates (SC), immunocastrates (IC) and entire males (EM) of one crossbreed (Pietrain x German Landrace), which were fattened with the same diet ad libitum. Piglets of the SC group were surgically castrated within the first week of life, IC were vaccinated twice with Improvac® at an age of 12 (first vaccination—V1) and 22 weeks (second vaccination—V2). Pigs were slaughtered in an experimental slaughter unit (Landesanstalt für Schweinezucht Boxberg, Boxberg, Germany) using their standard procedures. For dry-cured ham processing, pigs (26 or 27 weeks old, with an average weight of 121.7 kg, *n* = 36) of one slaughter batch were included, one ham per pig was processed, giving a total of 12 hams for every sex group. A day after slaughter, the hams were cut from the carcasses between the 6th and 7th lumbar vertebra, shaped into the prescribed form for Kraški pršut and weighed. Subcutaneous fat thickness of the green ham was measured under the femoral head and pHu was measured in the semimembranosus muscle (SM) using a MP120 pH meter (Mettler-Toledo GmbH, Schwarzenbach, Switzerland). 

Hams were submitted to a standard salting duration with dry salting for 12 days at 2–4 °C (first salting at 7 days and second salting 5 days later). After salting, the residual salt was removed and the hams were kept in a resting phase (4–6 °C and 70%–85% relative humidity (RH)) for 73 days. After this salt equilibration phase, the hams were dried for 87 days (at 20 °C and 60%–80% RH). Thereafter the open surface of the hams was coated with a mixture of pork fat, pepper and flour to prevent too strong desiccation, then, the hams were ripened for another 196 days, resulting in the final processing duration of 368 days. At each processing step, the hams were weighed in order to monitor processing losses. At the end of processing, samples were taken from the central part of the ham, vacuum packed and frozen at −20 °C until analyzed. 

### 2.2. Color Measurements

Objective color parameters (CIE L*, a*, b*) were measured using a Minolta Chroma Meter CR-300 (Minolta Co. Ltd, Osaka, Japan) on the green ham muscle, gluteus medius (GM), on dry product muscles, the biceps femoris (BF), semitendinosus (ST), semimembranosus (SM), and the subcutaneous fat layer. The chroma (C*) value was calculated as √(a*^2^ + b*^2^) and hue angle (h°) value as tan^−1^(b*/a*), which denote color saturation and taint, respectively.

### 2.3. Chemical Analyses

Muscle samples (BF, SM, ST) were trimmed of superficial connective and fat tissue and pulverized in liquid nitrogen with a laboratory mill (Grindomix GM200, Retsch GmbH and Co., Haan, Germany). Total nitrogen, non-protein nitrogen (NPN) and salt (NaCl) contents were chemically determined as described by [13]. Briefly, the content of NaCl was determined by potentiometric titration using DL53 General Purpose Titrator (Mettler Toledo, Schwarzenbach, Switzerland), the proteolysis index (PI) was calculated as a ratio between NPN and total nitrogen content, while intramuscular fat (IMF) and dry-matter content were determined by near-infrared spectral analysis (NIR Systems 6500 Monochromator, Foss NIR System, Silver Spring, MD, USA) using internal calibrations. Water activity was measured with the HygroPalm AW1 SET instrument (Rotronic, Bassersdorf, Germany) using Aw Quick mode.

Thiobarbituric reactive substances (TBARS) of the BF muscle were determined according to the method described by Lynch and Frei [14]. Briefly, 0.5 g of sample was homogenized with 10 mL of 0.15 M KCl and 0.1 mM BHT, and an aliquot (0.5 mL) mixed with 1% (*w*/*v*) of 2-thiobarbituric acid in 50 mM NaOH and 2.8% (*w*/*v*) trichloroacetic acid, and incubated for 10 min at 100 °C in a thermostatic heating block. After cooling to room temperature, the pink chromogen was extracted into n-butanol and its absorbance was measured spectrophotometrically at 535 nm (BioSpectrometer Fluorescence, Eppendorf, Hamburg, Germany). TBARS concentration was expressed on a wet basis as μg malondialdehyde (MDA)/kg.

### 2.4. Texture Profile Measurements

Instrumental texture was measured in the SM, ST and BF muscles as previously described [13,15]. Two 15 mm thick pieces were taken from each ham and the muscles were excised, trimmed of fat and connective tissue. Six samples of defined dimensions (length × width × height = 20 mm × 20 mm × 15 mm) were submitted to stress relaxation (SR) and texture profile analysis (TPA) using a texture analyzer (Ametek Lloyd Instruments, Ltd., Bognor Regis, UK) with a 50 kg load cell and a 50 mm diameter compression plate. The SR test consisted of compressing the samples perpendicularly in the fiber-bundle direction to 25% of their initial height (crosshead speed of 1 mm/s). During compression for 90 s (speed of 50 points/s) the force was recorded and force decay coefficient (Y90) was calculated as Y90 = (F0 − F90)/F0, where F0 (N) is the initial force and F90 (N) is the force recorded after 90 s of relaxation. In the TPA test, the samples were compressed twice to 50% of their original height at a crosshead speed of 1 mm/s and the following parameters were calculated: hardness (N), cohesiveness, gumminess (N), springiness (mm), chewiness (N) and adhesiveness (N*mm).

### 2.5. Sensory Analysis 

The sensory qualities of the dry-cured hams were assessed using the quantitative descriptive analysis method [16]. Thirty-seven attributes were chosen and defined according to the profiling procedure. Sample evaluation included: four appearance traits (fat, meat, color uniformity, marbling), thirteen odor traits (meat, fatty, smoky, acidic, sweet, bouillon-like, fermentation, yeast, sweat, manure, sharp, rancid, overall odor intensity), five texture attributes (hardness, gumminess, dryness, fibrousness, ease of fragmentation), fourteen taste/flavor traits (meat, fatty, smoky, sour, salty, sweet, bitter, bouillon-like, fermentation, yeast, sweat, manure, persistent, rancid) and overall sensory quality. The intensity of the descriptors was measured on a linear unstructured scale (0–10 cm) anchored at both extremes from “none” (on the left) to “very strong” (on the right). Overall sensory quality of the dry-cured hams was defined as the impression of the harmony of the examined attributes, with no or only slight intensity of negative notes. 

The individual samples of dry-cured hams (one slice, 1 mm thick) were placed in coded (3-digit numbers) plastic containers (200 mL) and covered with lids. A meat slicer was used to cut the samples and the thickness of the first slice of dry-cured ham was additionally verified by a Vernier caliper. The samples were presented to the assessors in random order at room temperature (21 ± 2 °C) and under white bulb light. Unsweetened tea (at the temperature of approximately 50 °C) and a piece of matzah were used as a taste neutralizer between samples. The order of the samples presented to the panelists was balanced to minimize possible carry-over effects between dry-cured ham samples. The evaluations were conducted during the morning and afternoon hours, with two sessions per each set of three samples from EM, IC and SC pigs. The assessment of the samples was performed in an accredited sensory laboratory, equipped with 10 individual testing booths (ISO 8589:2007 [17]). 

Ten trained and experienced assessors sensitive to boar compounds performed the profiling of the samples in two replications (ISO 8586:2012 [18]). Assessors were tested for their ability to qualitatively and quantitatively differentiate the odor of skatole and androstenone in low, high and very high concentrations on paper strips. Twenty individual results for each dry-cured ham sample were used for statistical analysis and interpretation of the sensory data according to the experimental design. 

### 2.6. Statistical Analysis

To determine the effect of male sex group on green ham traits, ham processing weight losses, chemical properties and instrumental texture measurements, the results were submitted to one-way analysis of variance with a male sex group as fixed effect using the Mixed procedure of SAS statistical software (SAS Institute Inc., Cary, NC, USA). In the case of sensory traits, repeated measures analysis was performed with the model including male sex group and session as fixed and panelist as random effects. To assess differences between groups, the least squares means (LSM) were compared using Tukey’s *t*-test. A cut-off *p*-value below 0.05 was considered significant.

## 3. Results

### 3.1. Green Ham Properties and Ham Processing Weight Loss

Table 1 presents the information on green ham properties and weight losses during the processing. There were no differences observed between EM, IC and SC in trimmed ham weight and pH value measured in SM muscle, whereas subcutaneous fat was significantly thicker in SC than in EM or IC hams (*p* < 0.0001). Color measurements exhibited differences for glycolytic GM but not oxidative GP muscle. IC had a higher L* value of GM muscle, i.e., lighter color than EM (*p* = 0.02), while SC had an intermediate position, and did not differ from EM or IC. Regarding processing weight loss, the hams from SC pigs exhibited significantly higher yields (*p* < 0.01) than IC or EM, which were similar in this respect.

### 3.2. Physical-Chemical Properties of Dry-Cured Ham Muscles

Table 2 provides the results of the physical-chemical analysis for three dry-cured ham muscles, outer SM and inner ST and BF. There was a significant effect of male sex group (*p* < 0.05) on the proximate composition of all three muscles, showing that at the end of processing, muscles of SC retained more water and less salt. Consequently, water activity was higher in SC than EM or IC in all three muscles. Ham muscles from SC also had higher fat and lower protein content. Considering the index of proteolysis (ratio of non-protein to total nitrogen), the effect of male sex group differed according to muscle. It was not affected in the outer SM muscle, it was higher in SC and IC than EM in the inner ST, and it was higher in SC than IC or EM in the inner BF muscle. 

### 3.3. Instrumental Texture Profile of Dry-Cured Ham Muscles

Instrumental measurements of the texture profile revealed the mechanical properties of dry-cured muscles and it can be observed that the majority of them were significantly affected by male sex group (Table 3). Based on texture profile traits depicted per muscle it can be summarized that dry-cured ham muscles from SC differ from IC and EM. Compared with EM and IC, SC dry-cured ham muscles are softer, less gummy, less springy and less chewy, but more adhesive. Dry-cured ham muscles of SC also have a higher stress relaxation coefficient, which denotes a more plastic structure.

### 3.4. Sensory Analysis of Dry-Cured Ham

Male sex group affected (*p* < 0.05) or tended to affect (*p* < 0.10) several sensory attributes of the examined dry-cured hams (Table 4). With regard to the appearance of the slices of ham, EM were leaner and less marbled than SC, with IC taking a somewhat intermediate position. With regard to the odor, EM had a slightly less meaty and bouillon-like odor (*p* < 0.10), but an intensely sharper odor with a strong sweat and manure taint. IC and SC were comparable with regard to odor. In the case of texture, EM and IC were similar (no significant differences) whereas SC exhibited significantly different texture from both IC and EM, i.e., it was softer, less gummy, less dry, less fibrous and the dry-cured ham slices had a more easily fragmentable texture. EM hams were different from SC and IC in the following taste and flavor attributes, i.e., meat, sweet, bouillon-like (lower), sweat, manure, persistence (higher) whereas IC hams were different from EM and SC in their saltiness (higher). The overall impression of sensory quality revealed that SC dry-cured ham were the most appreciated and EM dry-cured hams the least, and IC were in between, and different from both SC and EM.

## 4. Discussion

Due to their effect on dehydration, salt intake and biochemical changes during processing [10], green ham properties, which can be assessed without damage to further processing, were recorded. The main difference between male sex groups was in subcutaneous fat cover which was thicker in SC than EM or IC. As shown by meta-analytical studies, an absence of differences in subcutaneous fat thickness between EM and IC is generally observed [19,20]. Ham fat thickness is the principal factor affecting seasoning loss, with fatter hams exhibiting less and slower dehydration [21]. In agreement with this, lower processing loss was observed in SC than EM or IC hams (7% or 5%-point difference, respectively). In our recent study [22], we also observed a significant difference in processing weight loss between IC and EM, whereas in the present study, a 2%-point better yield in IC hams was not significant, but was consistent with insignificantly thicker fat cover and intramuscular fat of IC dry-cured ham muscles.

The observed physical-chemical properties of dry-cured ham muscles show that it was mainly SC that differed from EM and IC (which were similar in this respect). Higher water content and lower salt intake in SC than EM or IC corroborates the results for processing loss and the results in the literature on salt intake and water migration during processing, as monitored with computed tomography [23]. It is worth noting that due to lower dehydration, SC hams also had lower protein percentages than EM or IC. As a result, dry-cured ham muscle of SC presented higher water activity, which together with lower salt content may affect the proteolysis. It has been demonstrated that proteolysis is more pronounced in hams with reduced salt content or inversely, that protein breakdown is lower when salt concentration is higher [22,24,25]. High salt concentration inhibits muscle proteases responsible for the degradation of proteins to short peptides and free amino acids [26]. With regard to the index of proteolysis, the effect of male sex group differed according to the muscle. It was insignificant in SM muscle, which also exhibited lower PI values due to the fact that this muscle is exposed to air and salt and is thus submitted to more intense desiccation. In the case of ST and BF muscles, SC had higher PI than EM, whereas IC were SC-like in the ST muscle, and EM-like in the BF muscle. This inconsistency in IC response may indicate that IC behave differently from EM and SC in terms of proteolytic enzymes activity and protein breakdown. It has been suggested that EM have a higher degree of proteolysis than SC [27] and that higher androgenic potential (androstenone level) might be associated with increased proteolysis [28], which could not be confirmed in the present study. The only available relevant study including IC proteolytic potential [29] showed higher cathepsin B activity in green ham muscle of IC than SC, but showed the opposite trend for proteolysis (i.e., the highest PI was in SC hams). 

The instrumentally assessed texture of dry-cured ham muscles also showed that it was mainly SC that differed from EM and IC, in line with the differences in physical-chemical properties. Dry-cured ham texture is due to dehydration, which causes hardening of the product [30] or to proteolysis [31], which increases the softness as a result of the cleavage of structural proteins. The absence of important differences in the rheological parameters of EM and IC have been reported previously [22]. The instrumentally assessed texture was consistent with the sensory evaluation by the expert panel, and confirmed that with respect to texture, dry-cured hams from IC are EM-like, while both were different from SC. 

Visual assessment of dry-cured ham slices confirmed that EM are leaner than SC, and that IC were more EM-like with regard to fatness indicators, in line with results for the chemical composition of green ham or dry-cured ham. However, because of the potential interfering effect of boar taint presence in EM, the most important result of sensory analysis is the perception of odor, taste and flavor. Indeed, the results confirmed a clear, distinct profile for dry-cured hams from EM, while IC and SC exhibited similar sensory profiles. Significantly stronger perception of several sensory attributes (sweat, manure, sharp, persistent) that the literature describes for pork from EM [32], proves that boar taint did not diminish with processing and that panelists were able to detect the organoleptic defects in dry-cured hams from EM. Actually, 9 out of 12 EM hams presented androstenone levels above 1 µg/g while only 2 out of 12 hams presented skatole levels above 0.25 µg/g (data not shown), which are considered threshold levels for sensory perception. On the other hand, in hams from IC pigs the androstenone and skatole levels were below the detection level (0.24 and 0.03 µg/g liquid fat, respectively) [12]. Studies evaluating the effect of processing technologies on boar-tainted meat generally show that dry-curing does not eliminate its perception in the products [7,8]. It also seems that boar taint substances are neither degraded nor lost during the long dry-curing process [33].

## 5. Conclusions

The present investigation demonstrated that the quality of dry-cured ham is strongly affected by male sex group and that dry-cured ham from entire (uncastrated) males has sensory quality defects and also differs in other aspects that are important in dry-cured ham production (e.g., insufficient subcutaneous fat thickness). In the experimental conditions of the present study, the immunocastrates also produced hams with a fat thickness below the requirement for Kraški pršut PGI labelling, suggesting that to better meet the requirements for special products, the vaccination protocols (e.g., longer time delay between second vaccination and slaughter) and other management practices could be adapted or further optimized. Regarding the aptitude for processing into traditional dry-cured products, under the conditions of the present study, i.e., standard age and weight of pigs, lean type crossbreed, same diet, usual delay between effective immunocastration and slaughter, the immunocastrated pigs are more similar to entire males, however their main advantage is that they are free of boar taint, while the surgical castrates had the most appropriate raw hams for processing into traditional dry-cured products.

## Figures and Tables

**Table 1:** Effect of male sex group on green ham properties and ham processing weight loss.

	Male Sex Group				
Trait	EM	IC	SC	RMSE	*p*-Value
Ham trimmed, kg	11.1	11.2	11.8	0.947	0.1677
Fat thickness, mm	8.0 ^a^	9.2 ^a^	14.2 ^b^	2.619	<0.0001
SM muscle pHu	5.55	5.53	5.57	0.061	0.3246
GM muscle color					
L*	47.5 ^a^	50.1 ^b^	48.8 ^a,b^	2.181	0.0213
a*	9.8	9.8	9.5	1.429	0.8606
b*	6.1	6.7	6.4	0.9584	0.3932
c*	11.5	11.9	11.5	1.584	0.8096
H°	32.3	34.1	34.1	3.364	0.3260
GP muscle color					
L*	39.3	38.9	41.6	3.412	0.1293
a*	16.2	18.0	17.3	2.289	0.1998
b*	6.9	8.0	8.3	1.873	0.1792
c*	17.6	19.7	19.3	2.764	0.1917
H°	23.0	23.4	25.6	3.101	0.1055
Ham weight loss, %					
First salting	3.1 ^b^	2.8 ^a,b^	2.5 ^a^	0.347	0.0022
Second salting	1.5 ^b^	1.5 ^b^	1.0 ^a^	0.270	<0.0001
Resting	16.8 ^b^	16.1 ^b^	14.4 ^a^	1.234	0.0002
Drying	9.3 ^b^	9.0 ^b^	7.7 ^a^	0.724	<0.0001
Ripening	8.5 ^b^	7.8 ^b^	6.5 ^a^	0.723	<0.0001
Total	39.2 ^b^	37.2 ^b^	32.2 ^a^	2.669	<0.0001

Fat thickness was measured under the femoral head. SM semimembranosus; pHu denotes pH value measured 24 h post-mortem; GM gluteus medius; GP gluteus profundus; EM entire males; IC immunocastrates, SC surgical castrates, RMSE root-mean-square error. Values with different superscripts are significantly different (*p* < 0.05).

**Table 2:** Effect of male sex group on physical-chemical properties of dry-cured ham muscles.

	Male Sex Group				
Trait	EM	IC	SC	RMSE	*p*-Value
SM muscle					
Water (g/kg)	497.1 ^a,b^	488.6 ^a^	510.3 ^b^	16.9	0.0126
IMF (g/kg)	34.6 ^a^	40.7 ^a,b^	48.2 ^b^	8.9	0.0039
Salt (g/kg)	53.2 ^b^	51.2 ^a,b^	45.6 ^a^	5.1	0.0037
Proteins (g/kg)	399.6 ^a^	405.6 ^a^	382.1 ^b^	15.9	0.0031
PI, %	17.8	17.6	18.0	1.2	0.7330
aw	0.915 ^a^	0.917 ^a^	0.933 ^b^	0.010	0.0003
ST muscle					
Water (g/kg)	561.1	563.9	569.8	17.7	0.6600
IMF (g/kg)	45.8 ^a^	62.2 ^a^	86.3 ^b^	20.1	0.0001
Salt (g/kg)	58.8 ^a^	57.7 ^a^	49.3 ^b^	6.0	0.0009
Proteins (g/kg)	316.6 ^a^	302.4 ^b^	282.1 ^c^	13.5	<0.0001
PI, %	22.1 ^a^	24.6 ^b^	26.4 ^b^	1.9	<0.0001
a_w_	0.922 ^a^	0.926 ^a^	0.944 ^b^	0.011	<0.0001
BF muscle					
Water (g/kg)	596.7 ^a^	599.1 ^a^	617.3 ^b^	13.6	0.0014
IMF (g/kg)	21.6 ^a^	26.7 ^a,b^	32.5 ^b^	7.6	0.0070
Salt (g/kg)	64.5 ^a^	62.8 ^a^	55.5 ^b^	5.9	0.0017
Proteins (g/kg)	300.0 ^a^	293.6 ^a^	275.1 ^b^	1.6	<0.0001
PI, %	23.6 ^a^	24.4 ^a^	27.7 ^b^	1.6	<0.0001
a_w_	0.917 ^a^	0.922 ^a^	0.941 ^b^	0.010	<0.0001
TBARS (μg MDA/kg)	4.0	4.5	4.1	1.5	0.625

SM semimembranosus; ST semitendinosus; BF Biceps femoris; PI index of proteolysis; a_w_ water activity; TBARS thiobarbituric reactive substances; MDA malondialdehyde; EM entire males; IC immunocastrates, SC surgical castrates, RMSE root-mean-square error. Values with different superscripts are significantly different (*p* < 0.05).

**Table 3:** Effect of male sex group on measurements of texture profile of dry-cured ham muscles.

	Male Sex Group				
Trait	EM	IC	SC	RMSE	*p*-Value
SM muscle					
Hardness	128 ^b^	112 ^b^	67 ^a^	31	0.0001
Cohesiveness	0.54	0.50	0.53	0.08	0.5848
Gumminess	68 ^b^	56 ^b^	37 ^a^	17	0.0006
Springiness	4.7 ^b^	4.7 ^b^	4.1 ^a^	0.53	0.0103
Chewiness	323 ^b^	253 ^b^	161 ^a^	89	0.0005
Adhesiveness	−0.56 ^b^	−0.72 ^b^	−1.26 ^a^	0.40	0.0005
Y90	0.611 ^a,b^	0.601 ^a^	0.631 ^b^	0.022	0.0081
ST muscle					
Hardness	34 ^b^	31 ^a,b^	21 ^a^	11	0.0140
Cohesiveness	0.42 ^b^	0.39 ^a,b^	0.36 ^a^	0.06	0.0503
Gumminess	15 ^b^	13 ^a,b^	7 ^a^	7	0.0282
Springiness	4.3	4.4	3.7	0.8	0.0532
Chewiness	70 ^b^	54 ^a,b^	28 ^a^	35	0.0226
Adhesiveness	−2.2 ^a^	−2.4 ^a,b^	−3.0 ^b^	0.6	0.0105
Y90	0.690 ^a^	0.690 ^a^	0.713 ^b^	0.021	0.0132
BF muscle					
Hardness	46	52	40	12	0.0557
Cohesiveness	0.52 ^b^	0.53 ^b^	0.42 ^a^	0.70	0.0011
Gumminess	24 ^a,b^	29 ^b^	17 ^a^	10	0.0169
Springiness	4.3 b	4.1 ^b^	3.5 ^a^	0.48	0.0003
Chewiness	108 ^a,b^	127 ^b^	60 ^a^	54	0.0128
Adhesiveness	−2.7 ^b^	−2.6 ^b^	−3.4 ^a^	0.75	0.0026
Y90	0.694 ^a^	0.692 ^a^	0.728 b	0.023	0.0009

SM semimembranosus; ST semitendinosus; BF biceps femoris; Y90 stress relaxation parameter; EM entire males; IC immunocastrates, SC surgical castrates, RMSE root-mean-square error. Values with different superscripts are significantly different (*p* < 0.05).

**Table 4:** Effect of sex group on sensory traits of dry-cured hams.

	Male Sex Group				
Trait	EM	IC	SC	RMSE	*p*-Value
Appearance attributes					
Fat visually	3.8	3.7	3.2	1.3	0.0587
Meat visually	6.3 ^c^	5.4 ^b^	4.6 ^a^	1.3	<0.0001
Meat color uniformity	5.8	5.6	5.7	1.2	0.3950
Marbling visually	3.4 ^a^	3.8 ^a^	4.4 ^b^	1.5	0.0002
Odor attributes					
Meat	4.6	4.8	4.8	0.7	0.0739
Fatty	3.4	3.4	3.6	0.8	0.5055
Smoky	2.7	2.8	2.8	1.0	0.6830
Acidic	2.0	2.1	2.0	0.8	0.4956
Sweet	0.9	0.9	1.0	0.5	0.2040
Bouillon-like	1.6	1.7	1.8	0.7	0.0941
Fermentation	2.8	3.1	2.9	0.9	0.1856
Yeast	1.4	1.5	1.5	0.7	0.8739
Sweat	1.7 ^b^	0.4 ^a^	0.3 ^a^	0.7	<0.0001
Manure	0.7 ^b^	0.3 ^a^	0.2 ^a^	0.6	<0.0001
Sharp	2.4 ^b^	1.7 ^a^	1.6 ^a^	0.9	<0.0001
Rancid	0.9	0.9	0.8	0.6	0.2466
Overall odor intensity	5.1	5.1	4.9	0.9	0.4286
Texture attributes					
Hardness	4.2 ^b^	3.8 ^b^	2.8 ^a^	1.0	<0.0001
Gumminess	4.3 ^b^	4.1 ^b^	3.3 ^a^	1.1	<0.0001
Dryness	4.8 ^b^	4.5 ^b^	3.7 ^a^	1.1	<0.0001
Fibrousness	4.1 ^b^	4.0 ^b^	3.4 ^a^	1.2	<0.0001
Ease of fragmentation	5.8 ^a^	6.3 ^b^	6.9 ^c^	1.2	<0.0001
Taste and flavor attributes					
Meat	5.2 ^a^	5.6 ^b^	5.6 ^b^	0.7	<0.0001
Fatty	3.3	3.2	3.4	0.9	0.6251
Smoky	2.8	3.0	2.9	1.0	0.4418
Sour	2.2	2.5	2.3	0.7	0.0645
Salty	5.0 ^a^	5.4 ^b^	5.0 ^a^	0.9	0.0159
Sweet	0.8 ^a^	1.0 ^b^	1.0 ^b^	0.6	0.0027
Bitter	0.6	0.7	0.6	0.6	0.7524
Bouillon-like	1.7 ^a^	2.0 ^b^	1.9 ^a,b^	0.7	0.0312
Fermentation	2.6	3.0	2.8	1.0	0.0868
Yeast	1.4	1.5	1.3	0.8	0.3342
Sweat	2.8 ^b^	0.3 ^a^	0.3 ^a^	0.8	<0.0001
Manure	0.9 ^b^	0.2 ^a^	0.2 ^a^	0.6	<0.0001
Persistent	2.7 ^b^	0.8 ^a^	0.7 ^a^	0.9	<0.0001
Rancid	0.7	0.7	0.6	0.6	0.4085
Overall sensory quality	4.2 ^a^	5.9 ^b^	6.2 ^c^	0.9	<0.0001

EM entire males; IC immunocastrates, SC surgical castrates, RMSE root-mean-square error. Values with different superscripts are significantly different (*p* < 0.05).
